# Effect of Cutting Process Parameters on the Cutting Accuracy of Multilayer Surgical Gauze Using a Drum Cutting Unit

**DOI:** 10.3390/ma19214560

**Published:** 2026-10-29

**Authors:** Marcin Zastempowski, Andrzej Bochat, Maciej Gajewski, Maciej Janiec

**Affiliations:** 1 Faculty of Mechanical Engineering, Bydgoszcz University of Science and Technology, Al. Prof. S. Kaliskiego 7, 85-796 Bydgoszcz, Poland; andrzej.bochat@pbs.edu.pl (A.B.); maciej.gajewski@pbs.edu.pl (M.G.); 2 Faculty of Mechatronics, Kazimierz Wielki University in Bydgoszcz, J.K. Chodkiewicza 30 Street, 85-064 Bydgoszcz, Poland; maciej.j@ukw.edu.pl

**Keywords:** surgical gauze, cutting accuracy, drum cutting unit, cutting speed, cutting angle, multilayer material

## Abstract

**Highlights:**

**What are the main findings?**

- Transverse cutting (0°) yielded the highest accuracy with the lowest cut-spacing error (1.61%).
- Increasing the number of gauze layers from 3 to 8 raised the cut-spacing error by 15.5%.
- Cutting speed affected accuracy nonlinearly, with higher mean error values observed at 3.00 and 3.34 m·s^−1^.

**What are the implications of the main findings?**

- Transverse cutting at moderate speeds minimizes geometric deviations in dressing production.
- Process parameters must be adjusted based on layer count to ensure dimensional repeatability.
- Provides a technical basis for optimizing drum cutting unit operation in gauze manufacturing.

**Abstract:**

The aim of this study was to determine the effect of selected parameters of the machine-cutting process of multilayer surgical gauze on the accuracy of the spacing of successive cuts in a material roll intended for dressing production. The experiments were conducted on an original test stand equipped with a drum cutting unit. Three variants of the number of surgical gauze layers were analysed: 3, 4, and 8; four cutting angles: 0°, 5°, 10°, and 15°; and nine cutting speeds ranging from 0.66 to 3.34 m·s^−1^. The relative spacing error between successive cuts, *δ_L_*, was adopted as a measure of process accuracy and was expressed as the percentage deviation of the actual distance between cuts from the theoretical value. For each parameter configuration, the value of *δ_L_* was determined on the basis of five replicate measurements. The results showed that cutting accuracy varied with the cutting angle, the number of layers, and the cutting speed. The lowest error values were obtained during transverse cutting at an angle of 0°. The mean value of *δ_L_* increased from 1.61% at 0° to 2.91% at 15°. Increasing the number of layers also resulted in an increase in the error; the mean values were 2.25%, 2.33%, and 2.59% for 3-, 4-, and 8-layer gauze, respectively. The effect of cutting speed was nonlinear. The lowest mean error values were observed at intermediate cutting speeds, whereas higher mean error values were obtained at 3.00 and 3.34 m·s^−1^. The minimum value of *δ_L_*, equal to 1.20%, was obtained for 3-layer gauze at a cutting angle of 0° and a cutting speed of 1.33 m·s^−1^, whereas the maximum value, equal to 3.57%, was obtained for 8-layer gauze at a cutting angle of 15° and a cutting speed of 3.34 m·s^−1^. The results indicate that the highest cut-spacing accuracy can be achieved during transverse cutting of the material, with a lower number of layers and at moderate cutting speeds. The findings can be used to select the operating parameters of drum cutting units used in machines for the production of surgical gauze dressings.

## 1. Introduction

The production of medical devices constitutes an important and dynamically developing sector of modern industry. This also applies to disposable products and dressing materials, the manufacture of which requires high quality, dimensional repeatability, and high efficiency of technological processes. The economic importance of this sector is confirmed by data concerning the European medical technology market, whose value in 2024 was estimated at approximately EUR 170 billion, corresponding to approximately 26.4% of the global market and making Europe the second-largest medical technology market worldwide [1].

Dressing materials include both traditional solutions, such as gauze, bandages, and cotton wool, as well as increasingly advanced materials, including hydrogel, hydrogel-polymer, alginate, and multifunctional dressings. Despite the intensive development of modern dressing materials, gauze remains one of the basic materials used in medical practice due to its availability, ease of use, and ability to absorb fluids and wound exudate [2,3]. Cotton gauze also continues to be developed as a base material for new functional dressings, including through surface modification and the introduction of antimicrobial properties [3].

From an industrial perspective, not only the properties of the dressing material itself are important, but also the accuracy and repeatability of the technological processes used during its processing. In the case of surgical gauze, the production process includes, among other operations, the preparation and multiple folding of the material web, its transport, periodic cutting or incision, trimming to the required dimensions, and preparation of the finished elements for subsequent technological operations and packaging. The accuracy of the spacing of successive cuts is directly important for the geometric repeatability of the resulting elements and for the correct course of subsequent production operations. In the case of a drum cutting unit, the position of the cuts results from the interaction between the movement of the material and the movement of the cutting-unit components and may therefore depend on the kinematic parameters of the process as well as on the properties and number of layers of the material being cut.

The cutting of textile materials has been widely analysed in the literature. The studies include both conventional and automated knife-cutting systems, as well as laser, ultrasonic, and other technologies used in the textile industry [4]. It has been demonstrated, among other findings, that process conditions and blade condition affect the quality and precision of cutting multilayer flexible materials [5], whereas in laser processing, the material properties and technological process parameters play a key role [6].

Much less attention has been devoted to the machine cutting of surgical gauze. Previous studies by the authors concerned the cutting of this material using a drum cutting unit and included determination of the effects of cutting speed, material feed angle, and number of layers on the specific cutting resistance and specific cutting work [7]. Among other findings, the existence of a threshold speed enabling proper cutting of the material and the dependence of the energy parameters of the process on the cutting conditions were demonstrated. These studies made it possible to characterise the mechanical and energy-related aspects of the process; however, they did not address the accuracy of the spacing of successive cuts in a moving gauze web. In contrast to the previous study [7], the present work focuses on the dimensional accuracy and repeatability of the cutting process by analysing the relative spacing error between successive cuts, rather than the mechanical resistance and energy required to perform the cut.

Studies on the industrial cutting of multilayer textile materials have shown that the cutting process is strongly influenced by the mechanical properties of the material, blade geometry, and operating parameters of the cutting system [8].

The heterogeneous and stochastic microstructure of fibrous materials may also contribute to local variations in deformation and mechanical response during processing [9].

Cutting accuracy is of particular importance under industrial conditions because deviations of the actual distance between successive cuts from the assumed value may affect the dimensional repeatability of the manufactured elements and the correct execution of subsequent technological operations. No studies were found in the available literature that determined the effect of the operating parameters of a drum cutting unit on the accuracy of cut spacing during the cutting of multilayer surgical gauze. The literature search indicates that publications mainly concern the general technology of textile cutting, edge quality, cutting forces, and tool wear, whereas the issue of the accuracy of surgical gauze cutting using drum cutting units remains poorly investigated [4,5,6,7].

Previous studies have also shown that cutting speed and cutting angle influence the cutting force and the interaction between the blade and fibrous or textile materials [10,11]. Parameter-dependent effects have also been reported for non-contact cutting technologies. In multilayer textile laser cutting, process speed and material structure were found to be important factors determining cutting performance and quality [12]. The cutting behaviour of fibrous materials is additionally affected by their heterogeneous structure, mechanical properties, and the complex interaction between individual fibres and the cutting tool [13]. Recent studies on automated knife cutting of flexible sheet materials have further demonstrated that feed rate, blade loading, material fixation, and toolpath geometry can directly affect cutting quality and geometric repeatability [14]. Therefore, the aim of this study was to determine the effect of selected cutting process parameters—cutting speed, cutting angle, and number of material layers—on the cutting accuracy of multilayer surgical gauze using a drum cutting unit. The relative spacing error between successive cuts in relation to the required theoretical distance was adopted as a measure of accuracy. The obtained results are intended to expand knowledge of the machine-cutting process of surgical gauze and to provide a basis for the rational selection of the operating parameters of cutting units used in the industrial production of dressing materials.

## 2. Materials and Methods

### 2.1. Experimental Design and Test Program

For the purposes of the experimental study, three independent variables were adopted:

(a) cutting speed *v*,
(b) surgical gauze cutting angle *α*,
(c) number of surgical gauze layers *n*.

**Figure 1:**
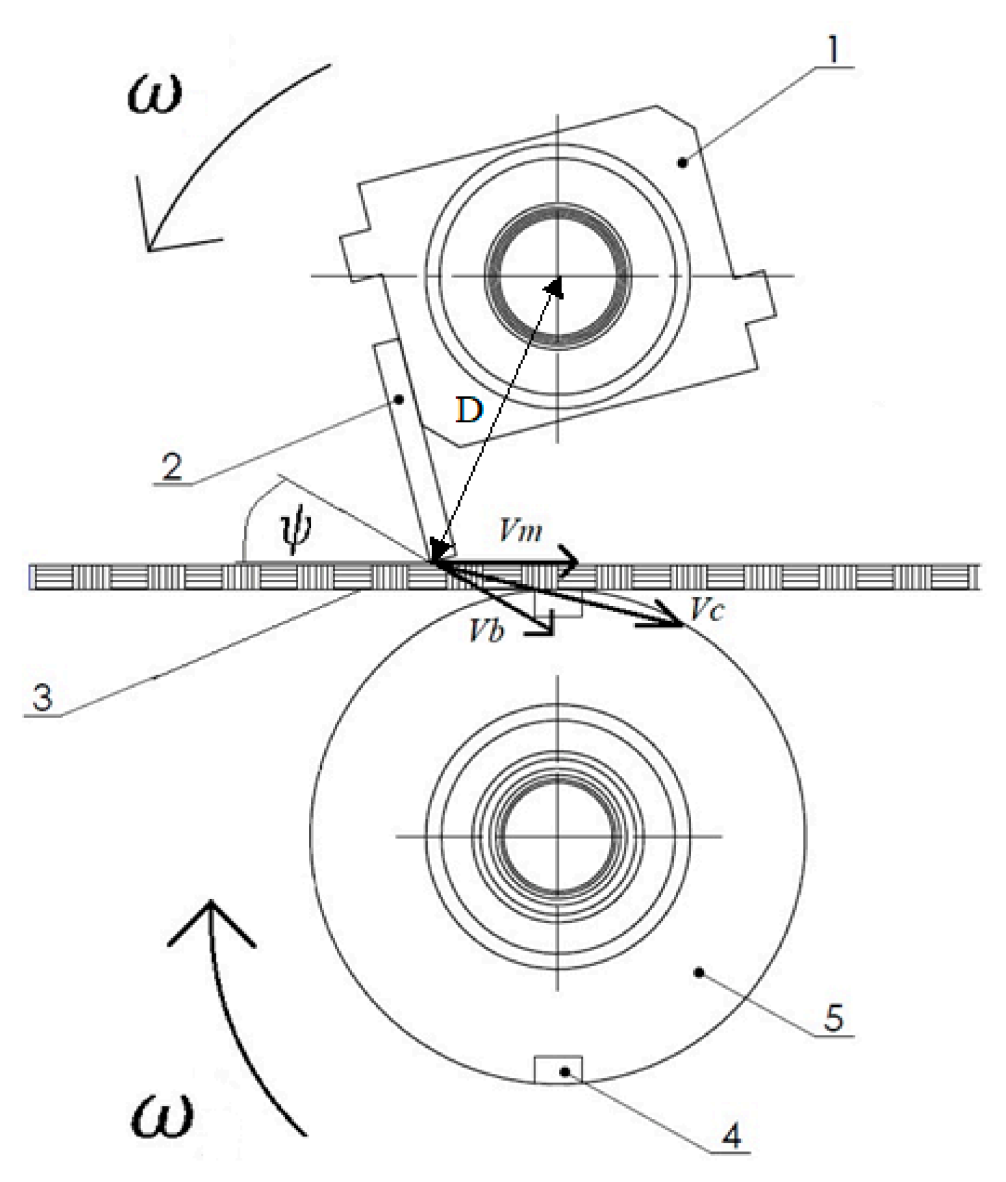
Diagram of surgical gauze cutting: 1—upper cutting drum, 2—cutting knife, 3—surgical gauze web, 4—counter-cutting strip, 5—lower cutting drum, *ω*—angular velocity of the upper and lower cutting drums, *ψ*—angle between the material feed velocity vector and the circumferential velocity vector of the cutting drum [7].

The cutting speed *v* was defined as the velocity of the knife blade at the moment of contact with the material being cut. The cutting-speed vector is the resultant of the circumferential velocity of the cutting knife and the linear velocity of material feeding. A kinematic diagram of the surgical gauze cutting process is shown in Figure 1.

A three-factor full factorial experimental design was adopted. The investigated factors were cutting speed (*v*), cutting angle (*α*), and number of surgical gauze layers (*n*). All combinations of the selected factor levels were tested, resulting in 9 × 4 × 3 = 108 experimental configurations. The investigated factors and their levels are summarized in Table 1.

The range of cutting speeds was selected on the basis of preliminary studies and the operating conditions of drum cutting units used in machines for the production of surgical gauze dressings [7]. The cutting angle *α* was defined as the angle between the cutting direction and the direction perpendicular to the movement of the gauze web. The value *α* = 0° corresponded to transverse cutting, whereas *α* = 5°, 10° and 15° corresponded to oblique cutting.

For each of the 108 experimental configurations, five replicate measurements were performed. The mean value obtained from the five replicates was subsequently used to determine the relative spacing error between successive cuts. Consequently, the experimental program comprised a total of 540 individual measurements. The analysis was descriptive and was based on comparisons of the mean values of *δ_L_* obtained for the investigated process-parameter configurations.

The selection of the number of layers resulted from practical requirements related to the production of multilayer surgical gauze dressings.

The relative spacing error between successive cuts *δ_L_* was adopted as the dependent variable and used as a measure of surgical gauze cutting accuracy. The relative spacing error between successive cuts was determined from the following relationship:

(1) $$\delta_{L}=\frac{|L_{T}-L_{R}|}{L_{T}}\cdot 100\%$$

where:

*L_T_*—theoretical distance between successive cuts,

*L_R_*—mean actual distance between successive cuts.

A lower value of *δ_L_* indicates greater accuracy of the spacing of successive cuts, whereas an increase in *δ_L_* indicates a greater deviation of the actual distance between cuts from the theoretical value.

### 2.2. Test Material

Surgical gauze with properties specified in the manufacturer’s material documentation was used as the test material. Type 13 gauze meeting the requirements of PN-EN 14079 was used in the experiments [15]. The basic properties of the material are presented in Table 2.

**Figure 2:**
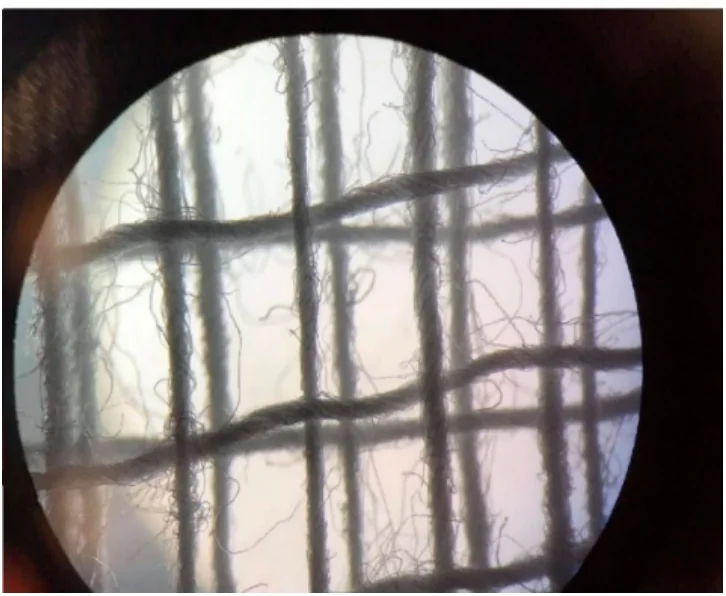
Microscopic image of the three-layer surgical gauze used in the experiments obtained using a ZEISS Stemi 508 (Carl Zeiss Microscopy GmbH, Jena, Germany) stereomicroscope at a magnification of 100×. The image illustrates the mutually displaced warp and weft systems of the individual gauze layers [7].

The surgical gauze used in the experiments had a woven structure with a plain weave. The material structure consisted of mutually perpendicular warp and weft threads made of cotton fibres [16]. The structure of the multilayer surgical gauze was observed using a ZEISS Stemi 508 stereomicroscope (Carl Zeiss Microscopy GmbH, Jena, Germany) at a magnification of 100×. An example image of the structure of the surgical gauze used in the experiments is shown in Figure 2.

Microscopic observations showed that the individual gauze layers did not overlap perfectly but were mutually displaced. The superposition of the displaced warp and weft systems resulted in a locally non-uniform multilayer architecture rather than a homogeneous material structure. This structural heterogeneity may influence local deformation, interlayer displacement, and the interaction between the material and the cutting blade during the cutting process.

### 2.3. Test Stand

An original test stand designed and constructed by the authors was used to carry out the experiments. The test stand enables investigation of the machine-cutting process of multilayer surgical gauze under controlled cutting speed, cutting angle, and number of material layers.

The main components of the test stand are:

- structural frame,
- rotary feeder with an unwinding and winding system for surgical gauze,
- drum cutting unit with a straight knife,
- drive systems and gear transmissions,
- control system.

**Figure 3:**
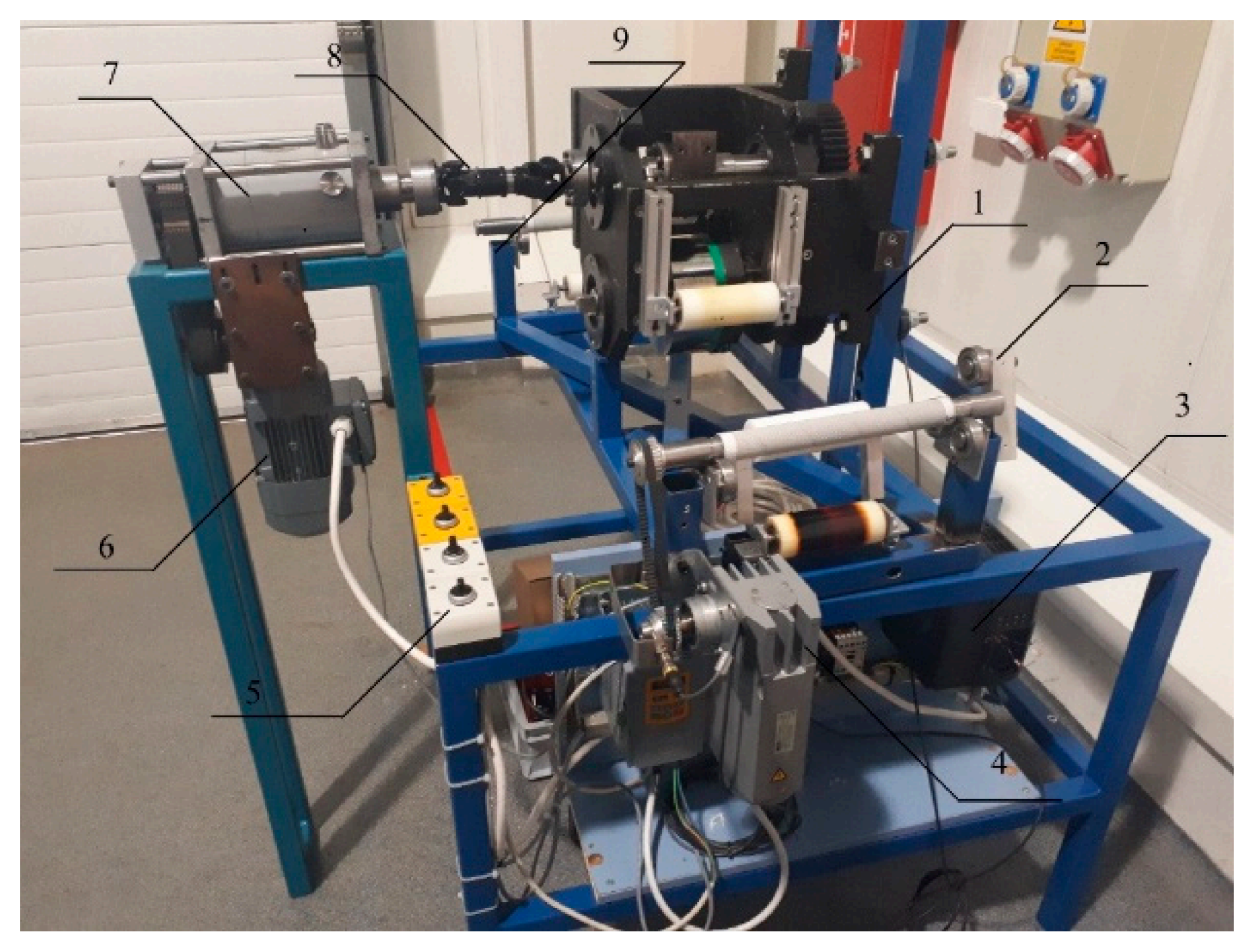
Test stand: 1—drum cutting unit, 2—winding unit, 3—control system, 4—electric motor with gearbox for the winder drive, 5—components for motor control and rotational speed adjustment, 6—electric motor with gearbox for the cutting-unit drive, 7—torque meter, 8—Cardan coupling, 9—material feeder frame [7].

A view of the test stand is shown in Figure 3.

The main element of the test stand is the drum cutting unit used to perform the surgical gauze cutting process. The unit is equipped with a straight knife mounted in a holder on the upper cutting drum. The knife was made of tool steel. Its design enables adjustment of its position relative to the counter-cutting edge by means of elongated mounting holes. This allows the geometry of the interaction between the knife and the counter-cutting element to be set correctly before the experiments are started.

The drive of the drum cutting unit is transmitted to the shaft of the upper drum, whereas the rotary motion from the upper drum to the lower drum is transmitted by means of a cylindrical gear transmission. This solution ensures synchronized motion of both elements of the cutting unit.

**Figure 4:**
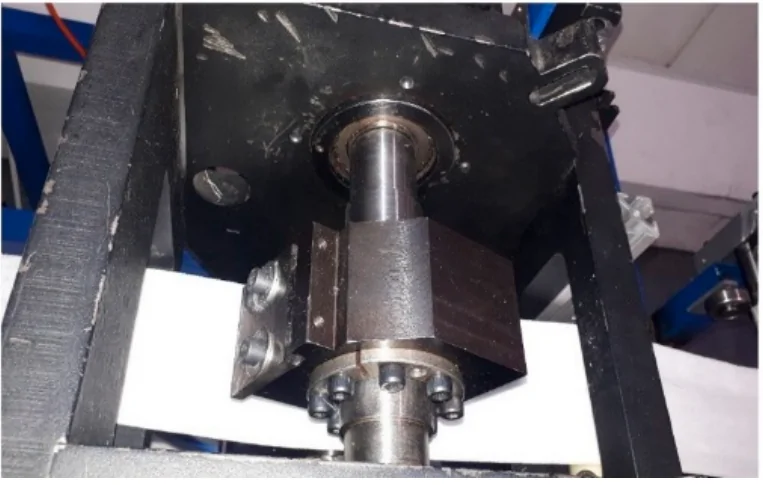
Upper cutting drum of the test stand [7].

A view of the upper cutting drum is shown in Figure 4.

The detailed design of the test stand and the operating principle of the drum cutting unit were also presented in the authors’ previous study concerning the mechanical and energy-related parameters of the surgical gauze cutting process [7]. In the present study, the test stand was used to evaluate the accuracy of the spacing of successive cuts in a multilayer surgical gauze web.

### 2.4. Research Methodology

The study evaluated the accuracy of the spacing of successive cuts made in a multilayer surgical gauze web. For this purpose, the material was cut at predetermined intervals corresponding to the assumed theoretical distance *L_T_*. The actual distance between two adjacent cuts was measured with the gauze web tensioned while maintaining its orientation along the warp and weft directions. For each configuration of process parameters, five replicate measurements of the distance between successive cuts were performed, after which the mean value *L_R_* was calculated. On this basis, the relative spacing error between cuts, *δ_L_*, was determined according to the relationship presented in Section 2.1.

The distance between successive cuts was measured using a Mitutoyo 500-706-11 ABSOLUTE Digimatic caliper (Mitutoyo Corporation, Kawasaki, Japan) with a measuring range of 0–150 mm, a resolution of 0.01 mm, and an accuracy of ±0.02 mm. The cutting angle was controlled using a Mitutoyo 187–552 digital universal protractor (Mitutoyo Corporation, Kawasaki, Japan) with a resolution of 0.01° and an accuracy of ±0.03°.

To minimize the influence of deformation of the flexible gauze web on the measurement results, the measurements were performed after stopping the test stand while the material remained tensioned between the unwinding and winding systems. During measurement, the gauze web was additionally supported on a flat surface to prevent sagging and local displacement of the material. The same measurement procedure was applied to all investigated gauze configurations.

**Figure 5:**
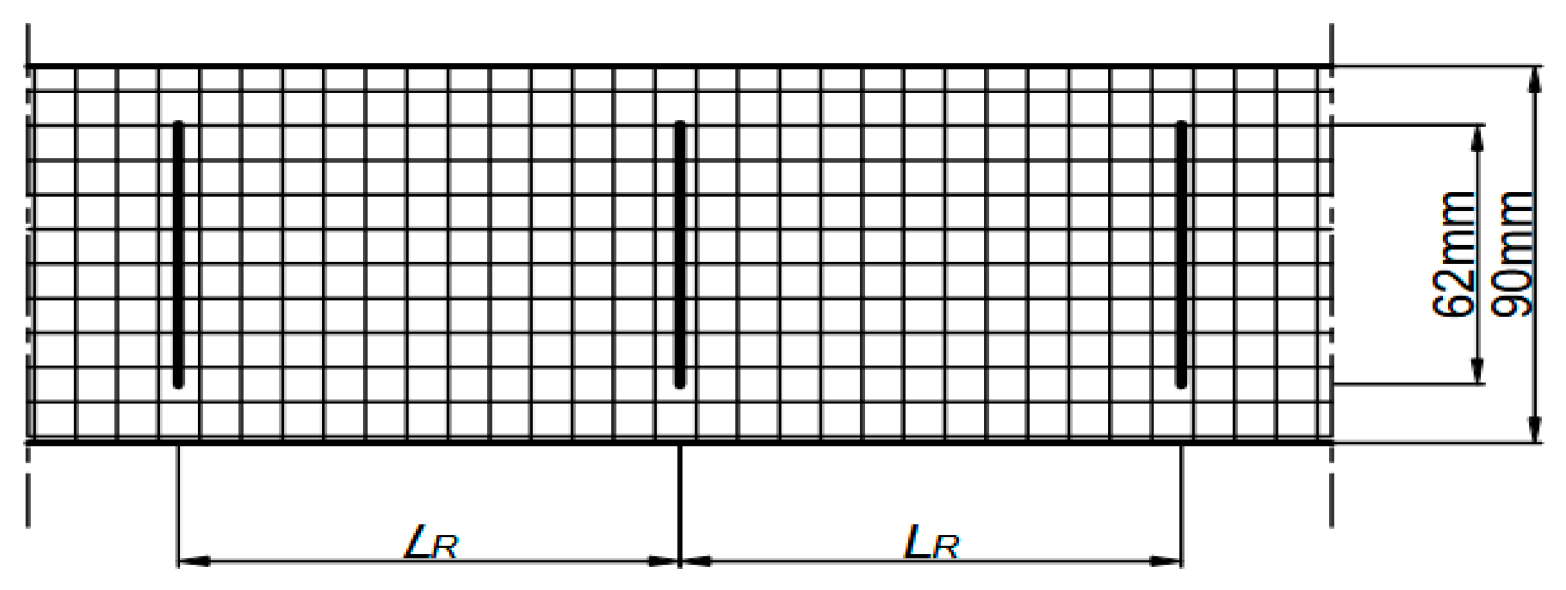
Diagram of transverse cutting of surgical gauze at *α* = 0°.

**Figure 6:**
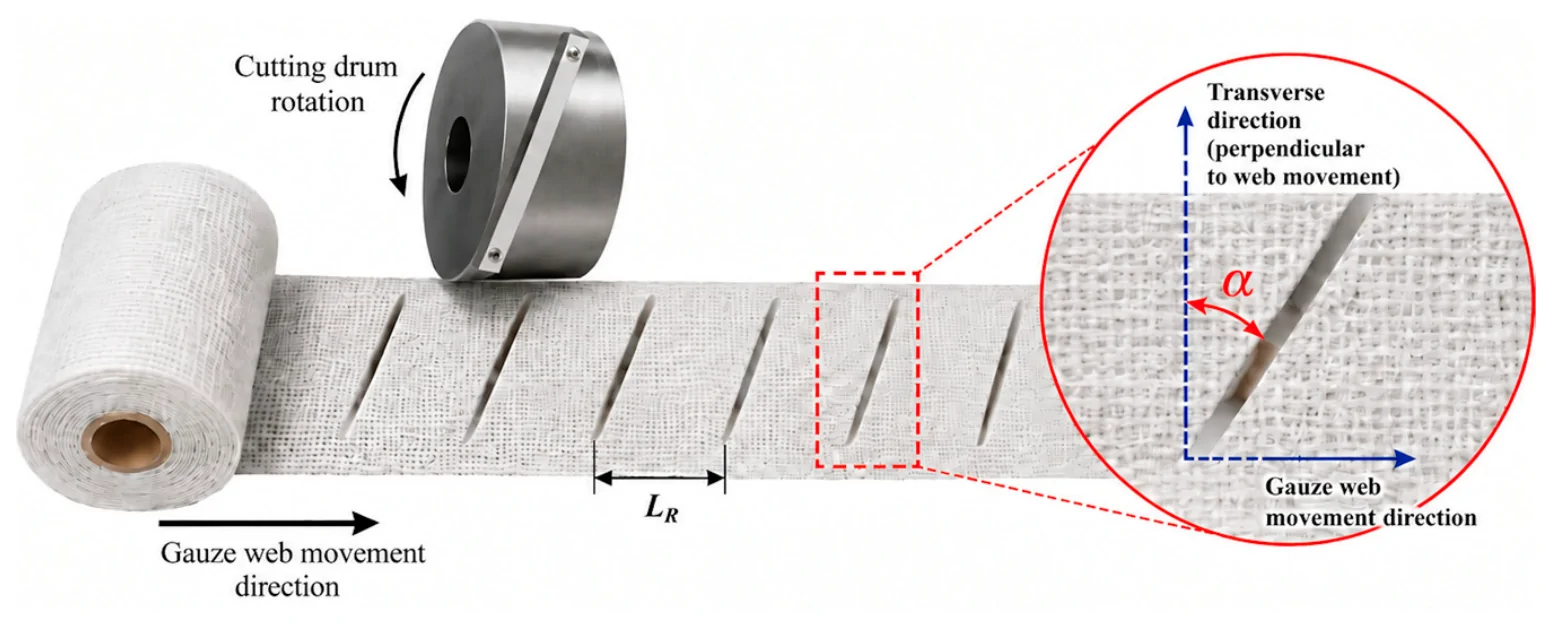
Schematic representation of oblique cutting of surgical gauze and definition of the cutting angle *α*.

Diagrams of transverse and oblique cutting of surgical gauze are shown in Figure 5 and Figure 6, respectively.

Before the experiments, multilayer rolls of surgical gauze were prepared in three configurations comprising 3, 4, and 8 layers, according to the adopted experimental program. The multilayer rolls were prepared using a gauze doubling machine, which enabled the required number of material layers to be obtained. Before testing, the material was checked for its condition and proper arrangement.

The test stand was equipped with two independent drive systems. The first system drove the drum cutting unit and enabled adjustment of its rotational speed. A SEW-Eurodrive WA20DR63L4/BR/TH electric motor (SEW-EURODRIVE GmbH & Co KG, Bruchsal, Germany) operating with a Siemens Micromaster 420 frequency inverter (Siemens AG, Munich, Germany) was used. The second drive system was responsible for the movement of the winder and thus for the linear feeding of the surgical gauze web. A BAUER-BG06-31/D07LA4-TOF-D electric motor (Bauer Gear Motor GmbH, Esslingen, Germany) operating with a Parker AC10 frequency inverter (Parker Hannifin Corporation, Mayfield Heights, OH, USA) was used. The cutting speed was defined as the resultant of the circumferential velocity of the cutting knife and the linear feeding velocity of the surgical gauze web. The rotational speed of the cutting drum and the feeding speed of the gauze were controlled independently using separate frequency inverters. During the experiments, the linear feeding velocity of the gauze web was kept constant, whereas the cutting speed was varied according to the adopted experimental program by changing the rotational speed of the cutting drum. During the cutting process, the gauze web was pulled from the unwinding roll by the driven winding system. The unwinder itself was not driven, and the winding system maintained continuous tension of the gauze web during its transport through the cutting unit. The same material-feeding procedure was applied to all investigated gauze configurations.

Before the measurements were started, the proper operation of the test stand, the setting of the drum cutting unit, and the operation of both drive systems were checked. A trial run of the cutting unit and the material transport system was then performed.

After setting the required number of gauze layers, cutting angle *α*, and cutting speed *v*, a series of cuts was made. For each combination of process parameters, five replicate measurements of the distance between adjacent cuts were performed. The measurement results were recorded, after which the mean actual spacing *L_R_* and the relative spacing error *δ_L_* were calculated.

Thus, the combination of 3 numbers of layers × 4 cutting angles × 9 cutting speeds resulted in a total of 108 experimental configurations.

## 3. Results

### 3.1. General Characteristics of the Results

Based on the conducted experiments, the values of the relative spacing error between successive cuts, *δ_L_*, were determined for 108 configurations of process parameters resulting from combinations of three numbers of surgical gauze layers, four cutting angles, and nine cutting speeds. Each analysed value of *δ_L_* was determined on the basis of the mean of five replicate measurements of the distance between successive cuts. Over the entire range of the experiments, the relative spacing error *δ_L_* ranged from 1.20% to 3.57%. The minimum value, equal to 1.20%, was obtained during cutting of 3-layer gauze at a cutting angle of *α* = 0° and a cutting speed of *v* = 1.33 m ⋅ s^−1^. The maximum value of *δ_L_* was observed during cutting of 8-layer gauze at a cutting angle of *α* = 15° and a cutting speed of *v* = 3.34 m ⋅ s^−1^.

The mean relative spacing error for all 108 configurations was 2.39%. The obtained results indicate that the value of *δ_L_* varied depending on all three analysed process parameters, with the largest relative differences observed in relation to the cutting angle.

The mean values of *δ_L_*, determined separately for the individual values of the analysed process parameters, are presented in Table 3.

The results indicate a systematic increase in the mean error value with an increasing number of material layers and an increasing cutting angle. The effect of cutting speed was more complex. The lowest mean value of *δ_L_* was obtained at a cutting speed of 1.33 m·s^−1^, whereas higher mean error values were observed at the two highest analysed cutting speeds, i.e., 3.00 and 3.34 m·s^−1^.

### 3.2. Effect of Cutting Angle on Cutting Accuracy

Analysis of the obtained results showed that the mean relative spacing error between successive cuts, *δ_L_*, varied with the cutting angle *α*.

The lowest error values were obtained during transverse cutting, i.e., at *α* = 0°. Increasing the cutting angle generally resulted in an increase in *δ_L_*, and consequently in a decrease in the accuracy of the spacing of successive cuts.

The mean value of *δ_L_*, calculated for all analysed cutting speeds and all numbers of layers, was 1.61% for *α* = 0°, 2.45% for *α* = 5°, 2.59% for *α* = 10°, and 2.91% for *α* = 15°. Thus, increasing the cutting angle from 0° to 15° resulted in an increase in the mean relative spacing error between cuts of approximately 81%.

The effect of the cutting angle was observed regardless of the number of material layers. For 3-layer gauze, the mean value of *δ_L_* increased from 1.50% at 0° to 2.76% at 15°. For 4-layer gauze, the corresponding values were 1.58% and 2.84%, whereas for 8-layer gauze they were 1.75% and 3.15%, respectively. Thus, in each of the analysed groups, the lowest error value was obtained for transverse cutting.

**Figure 7:**
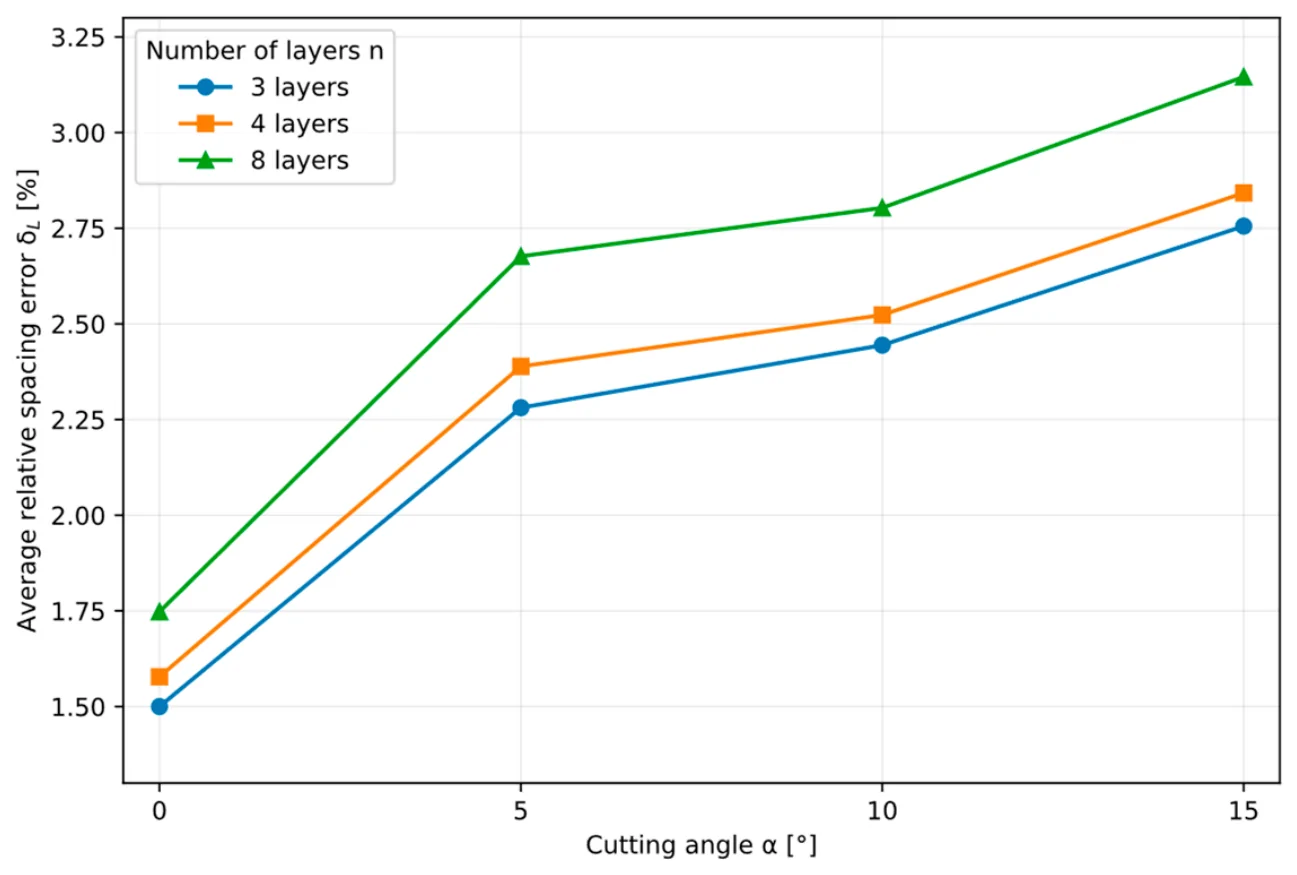
Average relative spacing error *δ_L_* as a function of the cutting angle *α* for 3-, 4-, and 8-layer surgical gauze.

Figure 7 presents the mean values of the relative spacing error *δ_L_* as a function of the cutting angle for the individual numbers of surgical gauze layers.

The relationships shown in Figure 7 indicate that changing the angle from 0° to 5° was associated with an increase in the mean error. A further increase in the angle to 10° and 15° resulted in a further, although less uniform, increase in *δ_L_*. This trend was similar for all three analysed numbers of layers.

The obtained results indicate that, from the point of view of the accuracy of the spacing of successive cuts, transverse cutting at *α* = 0° is the most favourable. As the cutting direction deviates further from the transverse direction, the error in reproducing the assumed spacing between successive cuts increases.

### 3.3. Effect of the Number of Surgical Gauze Layers on Cutting Accuracy

Analysis of the results showed that the relative spacing error between successive cuts *δ_L_*, increased with the number of surgical gauze layers. As the number of material layers increased, an increase in *δ_L_* was observed, indicating a decrease in the accuracy of the spacing of successive cuts.

The mean relative spacing error, calculated for all analysed cutting angles and cutting speeds, was 2.25% for 3-layer gauze, 2.33% for 4-layer gauze, and 2.59% for 8-layer gauze. Thus, increasing the number of layers from 3 to 8 resulted in an increase in the mean value of *δ_L_* of approximately 15.5%.

The consistency of this relationship across the investigated conditions is particularly noteworthy. For each of the 36 combinations of cutting angle and cutting speed, the lowest relative error value was obtained for 3-layer gauze, a higher value for 4-layer gauze, and the highest value for 8-layer gauze. It can therefore be concluded that the effect of the number of layers on cutting accuracy was consistent throughout the entire range of the experiments.

The effect of the number of layers was also evident when individual cutting angles were analysed. For transverse cutting, at *α* = 0°, the mean value of *δ_L_* increased from 1.50% for 3 layers to 1.75% for 8 layers. At *α* = 15°, the corresponding values were 2.76% and 3.15%. A similar trend was observed for cutting angles of 5° and 10°.

These relationships are shown in Figure 7, where, for all analysed cutting angles, the curve corresponding to 8-layer gauze lies above the curves corresponding to 3- and 4-layer gauze. The results therefore indicate that increasing the thickness of the multilayer web by increasing the number of layers makes it more difficult to maintain the assumed spacing between successive cuts.

From a practical point of view, this means that when cutting gauze with a greater number of layers, particular attention should be paid to the selection of the operating parameters of the cutting unit. This effect is especially evident at larger cutting angles, for which the relative error values are also higher.

### 3.4. Effect of Cutting Speed on Cutting Accuracy

Analysis of the results showed that the effect of cutting speed *v* on the relative spacing error between successive cuts, *δ_L_*, was nonlinear. In the range from 0.66 to 2.67 m·s^−1^, no monotonic increase or decrease in the error value was observed. Higher mean error values were observed at the two highest analysed cutting speeds, i.e., 3.00 and 3.34 m·s^−1^.

For all 108 configurations, the mean values of *δ_L_* were 2.29%, 2.29%, 2.20%, 2.25%, 2.36%, 2.30%, 2.36%, 2.66%, and 2.82% for cutting speeds from 0.66 to 3.34 m·s^−1^, respectively. The lowest mean error value was obtained at a cutting speed of 1.33 m·s^−1^, whereas the highest was obtained at 3.34 m·s^−1^.

A similar pattern of changes was observed for all analysed numbers of material layers. For 3-layer gauze, the mean value of *δ_L_*, calculated for the four cutting angles, was 2.06% at 1.33 m·s^−1^ and increased to 2.63% at 3.34 m·s^−1^. For 4-layer gauze, the corresponding values were 2.14% and 2.74%, whereas for 8-layer gauze they were 2.39% and 3.08%. This corresponds to an increase in the mean error value between 1.33 and 3.34 m·s^−1^ of approximately 27.6%, 28.3%, and 28.8% for 3-, 4-, and 8-layer gauze, respectively.

**Figure 8:**
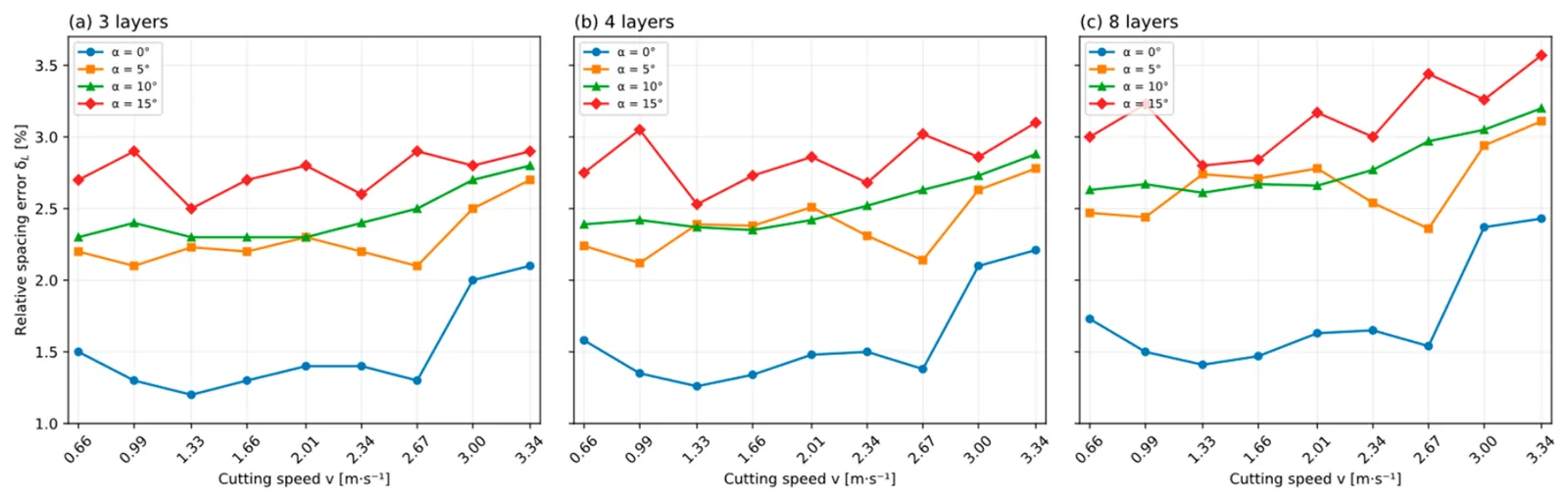
Relative spacing error *δ_L_* as a function of cutting speed *v* for multilayer surgical gauze at different cutting angles.

The relationship between the relative spacing error *δ_L_* and cutting speed for the individual cutting angles is shown in Figure 8.

Analysis of the curves shown in Figure 8 indicates that the effect of cutting speed also depended on the cutting angle. For *α* = 0°, the values remained relatively low over a wide range of cutting speeds, whereas higher error values were observed at 3.00–3.34 m·s^−1^. At *α* = 15°, the error values were higher throughout the entire range of the experiments, with the highest values observed at the maximum cutting speed.

In Figure 8a, corresponding to 3-layer gauze, the lowest values of *δ_L_* were obtained for transverse cutting and intermediate cutting speeds. As the cutting angle increased to 5°, 10°, and 15°, the error values increased, with the largest deviations observed at 15°. Similar relationships were found for 4-layer and 8-layer gauze, as shown in Figure 8b,c. At the same time, the overall level of *δ_L_* increased with the number of layers, confirming the previously discussed effect of the number of material layers on cutting accuracy.

## 4. Discussion

The obtained results therefore do not indicate that increasing the cutting speed causes a linear deterioration in accuracy. Within the investigated range, there is an intermediate range of cutting speeds in which the relative error values remain relatively low, whereas higher relative error values were observed at the highest cutting speeds.

The obtained results indicate that the cutting accuracy of multilayer surgical gauze depends on the conditions under which the process is carried out. Among the analysed parameters, the largest relative change in the mean spacing error was observed for the cutting angle. Increasing the angle from 0° to 15° resulted in an increase in the mean value of *δ_L_* from 1.61% to 2.91%, i.e., by approximately 81%. During transverse cutting, the cutting direction is perpendicular to the gauze-web movement, whereas oblique cutting introduces an additional longitudinal component of the knife–material interaction. In a flexible multilayer structure, this may increase the susceptibility of the material to local displacement during the cutting process. This result indicates that the geometry of the relative motion between the material web and the cutting element is of fundamental importance for maintaining the required repeatability of the positions of successive cuts.

The importance of geometry and the kinematic parameters of the process is consistent with the general principles of industrial cutting of textile materials, in which process accuracy depends both on the material properties and on the manner in which the material is fed, spread, and guided during cutting [4]. The literature on cutting flexible materials also indicates that the quality and precision of the process may depend on the operating conditions of the tool and its condition [5].

Increasing the number of gauze layers from 3 to 8 resulted in a systematic increase in the relative spacing error. The mean values of *δ_L_* were 2.25%, 2.33%, and 2.59% for 3-, 4, and 8-layer gauze, respectively. Importantly, for each analysed combination of cutting angle and cutting speed, the lowest error values were obtained for 3-layer gauze and the highest for 8-layer gauze.

The observed effect may be associated with the flexible and heterogeneous architecture of the multilayer gauze. As shown in Figure 2, the warp and weft systems of the individual layers are mutually displaced and do not form a geometrically uniform stack. With an increasing number of layers, the material system may therefore become more susceptible to local compression, relative displacement, and interlayer slip during transport and contact with the knife. These effects may contribute to deviations in the position of successive cuts. However, these mechanisms were not measured directly in the present study and should therefore be regarded as a physically plausible interpretation of the observed trend.

The observed relationship is consistent with broader findings concerning the industrial cutting and trimming of multilayer textile materials, for which the structure and arrangement of the material layers may affect process behaviour [4,17]. Studies on the mechanical cutting of multilayer flexible materials also indicate the importance of interactions between the material and the blade, as well as changes in cutting conditions, for the resulting process quality [5].

A different relationship was observed between cutting accuracy and cutting speed. In the range from 0.66 to 2.67 m·s^−1^, no monotonic trend in *δ_L_* was observed. The lowest mean error value was obtained at 1.33 m·s^−1^, whereas higher mean error values were observed at the highest investigated cutting speeds, i.e., 3.00 and 3.34 m·s^−1^. At the highest cutting speeds, dynamic effects associated with material transport and synchronization of the drive systems may become more important. Small variations in web tension, feed speed, or relative motion between the gauze and the cutting drum may therefore contribute to increased deviations in cut spacing. These effects were not measured directly and require further experimental verification.

This indicates that increasing process efficiency by increasing cutting speed may, above a certain range, lead to a deterioration in the accuracy of the spacing of successive cuts. Similarly, in other textile-processing technologies, proper selection of process parameters is one of the basic conditions for obtaining the required quality [6,18].

A particularly interesting aspect is the comparison of the present results with the authors’ previous study [7]. That study demonstrated that cutting speed, feed angle, and number of layers affect the mechanical and energy-related characteristics of the process. With increasing cutting speed, a tendency toward a decrease in specific cutting resistance was observed, and over a substantial part of the investigated range, a decrease in specific cutting work was also found [7]. The present results complement these observations by showing that an increase in cutting speed does not necessarily lead to a simultaneous improvement in all technological parameters of the process. At the highest analysed cutting speeds, an increase in the cut-spacing error was observed, even though the previous study indicated favourable changes in some energy-related parameters [7]. This means that the selection of the operating conditions of a drum cutting unit should be multi-criteria in nature and should take into account both process energy consumption and the required geometric accuracy of the product.

The results of the present study therefore extend the previous characterization of the surgical gauze cutting process. Previous studies on textile materials have focused mainly on cutting technologies, tool–material interaction forces, blade wear, edge quality, and optimization of technological parameters [4,5,6,19]. However, no studies were found describing the effect of cutting speed, cutting angle, and number of layers on the accuracy of the periodic spacing of cuts made by a drum cutting unit in multilayer surgical gauze.

From a practical manufacturing perspective, the relative spacing error considered in this study should primarily be interpreted as an indicator of process accuracy and dimensional repeatability rather than as a direct measure of the clinical performance of surgical gauze. Increased cut-spacing error may result in greater dimensional variability of the manufactured elements and may adversely affect subsequent operations such as folding, positioning, and packaging. If the resulting dimensions exceed the allowable manufacturing tolerances, this may increase the risk of product non-conformity and material waste. The present study did not evaluate functional medical properties such as absorbency, sterility, or clinical performance; therefore, no direct relationship between the measured cut-spacing error and the medical effectiveness of the gauze should be inferred.

The obtained results also indicate directions for further research on the cutting process of multilayer surgical gauze. It would be reasonable to extend the analyses to include the effects of material-web tension, mutual displacement of individual layers, blade wear, and synchronization between the material-feeding speed and the motion of the cutting drum. Such studies may enable a more detailed determination of the mechanisms responsible for the increase in cut-spacing error observed at higher numbers of layers, larger cutting angles, and the highest process speeds.

## 5. Conclusions

The present study demonstrated that the dimensional accuracy of multilayer surgical gauze cutting using a drum cutting unit is affected by the cutting angle, the number of material layers, and the cutting speed. The main findings can be summarized as follows:

1. Transverse cutting *α* = 0° provided the highest cut-spacing accuracy. Increasing the cutting angle to 15° increased the mean relative spacing error from 1.61% to 2.91%.
2. Increasing the number of gauze layers reduced cutting accuracy. The mean relative spacing error increased from 2.25% for 3-layer gauze to 2.59% for 8-layer gauze, and this trend was observed throughout the investigated range of cutting angles and speeds.
3. The effect of cutting speed was nonlinear. No monotonic trend was observed between 0.66 and 2.67 m·s^−1^, whereas higher mean error values were obtained at 3.00 and 3.34 m·s^−1^.
4. The lowest relative spacing error, 1.20%, was obtained for 3-layer gauze cut transversely at 1.33 m·s^−1^, whereas the highest value, 3.57%, occurred for 8-layer gauze cut at 15° and 3.34 m·s^−1^.
5. The results indicate that the operating parameters of drum cutting units should be selected by considering both process efficiency and dimensional accuracy. In particular, transverse cutting, a lower number of layers, and moderate cutting speeds favour improved geometric repeatability.
6. Further research should address the effects of material-web tension, interlayer displacement, blade wear, and synchronization between gauze feeding and cutting-drum motion in order to better explain the mechanisms responsible for cut-spacing deviations.

## Figures and Tables

**Table 1:** Factors and levels used in the experimental design.

Factor	Symbol	Unit	Levels
Cutting speed	*v*	m ⋅ s^−1^	0.66, 0.99, 1.33, 1.66, 2.01, 2.34, 2.67, 3.00, 3.34
Cutting angle	*α*	°	0, 5, 10, 15
Number of gauze layers	*n*	-	3, 4, 8

**Table 2:** Characteristics of the surgical gauze used in the experiments [7].

Parameter	Unit	Testing Method	Type of Gauze
			13 Threads/cm^2^
number of threads in the warp	threads/100 mm	PN-EN 14079	70 ± 4
number of threads in the weft	threads/100 mm		60 ± 4
surface weight	g⸱m^−2^		≥17
breaking strength in the warp	N/50 mm		≥35
breaking strength of the weft	N/50 mm		≥20
length of a roll of gauze	m.	-	≥2500 ± 10

**Table 3:** Mean values of the relative spacing error between cuts, *δ_L_*, for the analysed process parameters.

Parameter	Parameter Value	Mean *δ_L_* [%]
Number of layers *n*	3	2.25
	4	2.33
	8	2.59
Cutting angle *α*	0°	1.61
	5°	2.45
	10°	2.59
	15°	2.91
Cutting speed *v* [m·s^−1^]	0.66	2.29
	0.99	2.29
	1.33	2.20
	1.66	2.25
	2.01	2.36
	2.34	2.30
	2.67	2.36
	3.00	2.66
	3.34	2.82

## Data Availability

The original contributions presented in this study are included in the article. Further inquiries can be directed to the corresponding author.
